# Metabolic Fate of the Carboxyl Groups of Malate and Pyruvate and their Influence on δ^13^C of Leaf-Respired CO_2_ during Light Enhanced Dark Respiration

**DOI:** 10.3389/fpls.2016.00739

**Published:** 2016-06-03

**Authors:** Marco M. Lehmann, Frederik Wegener, Matti Barthel, Veronica G. Maurino, Rolf T. W. Siegwolf, Nina Buchmann, Christiane Werner, Roland A. Werner

**Affiliations:** ^1^Laboratory of Atmospheric Chemistry, Paul Scherrer InstituteVilligen, Switzerland; ^2^Institute of Agricultural Sciences, ETH ZurichZurich, Switzerland; ^3^Ecosystem Physiology, University of FreiburgFreiburg, Germany; ^4^Plant Molecular Physiology and Biotechnology Group, Institute of Developmental and Molecular Biology of Plants, Heinrich Heine University and Cluster of Excellence on Plant Sciences (CEPLAS)Düsseldorf, Germany

**Keywords:** fumarase, LEDR, malic acid, malic enzyme, pyruvic acid, respiration, stable carbon isotopes, TCA cycle

## Abstract

The enhanced CO_2_ release of illuminated leaves transferred into darkness, termed “light enhanced dark respiration (LEDR)”, is often associated with an increase in the carbon isotope ratio of the respired CO_2_ (δ^13^C_LEDR_). The latter has been hypothesized to result from different respiratory substrates and decarboxylation reactions in various metabolic pathways, which are poorly understood so far. To provide a better insight into the underlying metabolic processes of δ^13^C_LEDR_, we fed position-specific ^13^C-labeled malate and pyruvate via the xylem stream to leaves of species with high and low δ^13^C_LEDR_ values (*Halimium halimifolium* and *Oxalis triangularis*, respectively). During respective label application, we determined label-derived leaf ^13^CO_2_ respiration using laser spectroscopy and the ^13^C allocation to metabolic fractions during light–dark transitions. Our results clearly show that both carboxyl groups (C-1 and C-4 position) of malate similarly influence respiration and metabolic fractions in both species, indicating possible isotope randomization of the carboxyl groups of malate by the fumarase reaction. While C-2 position of pyruvate was only weakly respired, the species-specific difference in natural δ^13^C_LEDR_ patterns were best reflected by the ^13^CO_2_ respiration patterns of the C-1 position of pyruvate. Furthermore, ^13^C label from malate and pyruvate were mainly allocated to amino and organic acid fractions in both species and only little to sugar and lipid fractions. In summary, our results suggest that respiration of both carboxyl groups of malate (via fumarase) by tricarboxylic acid cycle reactions or by NAD-malic enzyme influences δ^13^C_LEDR_. The latter supplies the pyruvate dehydrogenase reaction, which in turn determines natural δ^13^C_LEDR_ pattern by releasing the C-1 position of pyruvate.

## Introduction

Transferring light-acclimated leaves into darkness lead to rapid changes in various metabolic processes, causing halt of any photosynthetic activity and increased respiration rates (Florez-Sarasa et al., 2012; Griffin and Turnbull, 2012). During such light–dark transitions, leaves show enhanced O_2_ consumption (Stone and Ganf, 1981; Azcon-Bieto et al., 1983) concurrently with enhanced CO_2_ release (Hill and Bryce, 1992; Atkin et al., 1998). The extent of this increasing CO_2_ emissions after darkening, defined as “light enhanced dark respiration (LEDR)”, depends on light intensity of the previous photoperiod and lasts about 20–30 min, with a maximum CO_2_ release within the first 5 min (Atkin et al., 1998; Griffin and Turnbull, 2012). LEDR does not interfere with the post-illumination burst, which occurs within <20 s after darkening and is related to photorespiratory glycine oxidation (Atkin et al., 1998).

During LEDR the carbon isotope ratio of leaf-respired CO_2_ (δ^13^C_LEDR_) is also changing, showing a strong initial increase which is followed by a progressive decrease (Werner et al., 2009; Wegener et al., 2010; Lehmann et al., 2016). Such a δ^13^C_LEDR_ pattern was also observed under natural conditions in a grassland ecosystem (Barbour et al., 2011), demonstrating that this phenomenon has also potential relevance at higher scales. δ^13^C_LEDR_ is also relevant for cases when leaf dark-respired CO_2_ is sampled from light-acclimated leaves. Many field (Prater et al., 2006; Sun et al., 2009; Dubbert et al., 2012) and laboratory experiments (Wegener et al., 2010; Lehmann et al., 2015, 2016) have shown that δ^13^C_LEDR_ is often less negative compared to respiratory substrates. The ^13^C enrichment in leaf-respired CO_2_ during LEDR progressively increases during daytime, which has been shown to be strongly related to the cumulative assimilation of CO_2_ (Hymus et al., 2005). Moreover, δ^13^C_LEDR_ may retain information of plant internal carbon allocation and was observed to differ among plant functional groups (Wegener et al., 2010, 2015): evergreen, slow-growing or aromatic species showed a very high increase in δ^13^C_LEDR_ during the day (up to 14.8‰), while fast-growing, non-aromatic herbaceous species showed no or a small increase in δ^13^C_LEDR_ (Lehmann et al., 2016). However, although leaf dark-respired CO_2_ is a potential and easy accessible indicator for leaf internal metabolic processes, high-resolution measurements of δ^13^C_LEDR_ are scarce (Werner et al., 2009; Jardine et al., 2014). Also the interpretation of this data is still difficult, since its respiratory substrates and associated enzymatic reactions are not fully understood so far (Werner and Gessler, 2011; Ghashghaie and Badeck, 2014).

δ^13^C_LEDR_ may depend on the heterogeneous intramolecular isotope distribution within respiratory substrates (e.g., sugars). For instance, the C-3 and C-4 positions of glucose are enriched in ^13^C compared to other positions of the same molecule (Rossmann et al., 1991; Gilbert et al., 2012), probably due to an equilibrium isotope effect of the aldolase reaction (Gleixner and Schmidt, 1997). On the one hand, ^13^C enriched positions of glucose molecules broken down during glycolysis are relocated to the C-1 position of pyruvate molecules and can be released as CO_2_ by the mitochondrial pyruvate dehydrogenase (mtPDH) reaction in the dark, influencing δ^13^C_LEDR_. Additionally, the chloroplast pyruvate dehydrogenase (cpPDH) reaction can consume photosynthetically produced pyruvate in the light (Tovar-Mendez et al., 2003). On the other hand, ^13^C depleted positions of glucose molecules relocated in the acetyl-CoA residue (C-2 and C-3 position of pyruvate) can be used in dark for respiration or for biosynthesis of various substrates such as amino acids, lipids, or organic acids (Tcherkez et al., 2009). Leaf feeding studies showed that the extent of ^13^CO_2_ respiration from ^13^C-1 pyruvate was higher than from pyruvate molecules labeled in other positions (Tcherkez et al., 2005, 2009). Other studies indicated that species-specific differences in ^13^CO_2_ respiration from ^13^C-1 pyruvate might explain the species-specific differences in δ^13^C_LEDR_ (Priault et al., 2009; Wegener et al., 2010).

Furthermore, also other respiratory substrates are expected to have a significant influence on δ^13^C_LEDR_. A study by Gessler et al. (2009) showed a clear malate breakdown shortly upon darkening in *Ricinus communis*, estimating that malate decarboxylation represents 22% of the respired CO_2_ during LEDR. The role of malate during LEDR was supported by a more recent study from Lehmann et al. (2015) who identified malate as an important and relatively ^13^C enriched respiratory substrate in *Solanum tuberosum*. The biochemical link between malate and δ^13^C_LEDR_ was explained with an anaplerotic flux via the PEPC reaction that replenishes TCA cycle intermediates, which are withdrawn for biosynthetic use (Werner et al., 2011; Lehmann et al., 2015). This is in line with other studies, suggesting that organic acids such as malate derived from PEPC may accumulate in leaves under illumination due to inhibition of enzymatic reactions in the mitochondrion such as fumarase, NAD-dependent isocitrate dehydrogenase (mtIDH), and 2-oxoglutarate dehydrogenase (OGDH) (Werner et al., 2011; Eprintsev et al., 2016), causing an open and only partially active TCA cycle (Hanning and Heldt, 1993; Tcherkez et al., 2005; Araújo et al., 2012). Thus, a rapid breakdown of malate via TCA recycling in the dark may be in support of enhanced CO_2_ release during LEDR and simultaneous increases in δ^13^C_LEDR_, but also other individual organic acids might be involved (Lehmann et al., 2016). In addition, malate derived from PEPC via oxaloacetate and the malate dehydrogenase reaction (MDH) is expected to be enriched in ^13^C at the C-4 position compared to other molecule positions (Melzer and O’Leary, 1987; Savidge and Blair, 2004), since PEPC fixes HCO_3_^-^ with a net isotope fractionation of -5.7‰ (with respect to dissolved CO_2_; Farquhar, 1983). Thus, decarboxylation of this malate molecule position in the dark by the previously light-inhibited mitochondrial NAD-ME reaction, as observed in *Hordeum vulgare* protoplasts (Hill and Bryce, 1992; Igamberdiev et al., 2001), might also contribute to the observed increase in δ^13^C_LEDR_ (Barbour et al., 2007; Gessler et al., 2009; Werner et al., 2011; Tcherkez et al., 2012), although NAD-ME might fractionate against ^13^C by about 20‰ as observed in *Crassula* plants (Grissom et al., 1987). Direct experimental evidence for respiration of malate is still missing and thus the respiratory and metabolic fate of the carboxyl groups (C-1 and C-4 position) of malate during light–dark transitions and their influence on δ^13^C_LEDR_ remains to be proven.

Here, we hypothesize that δ^13^C_LEDR_ is determined by decarboxylation of malate and pyruvate, but we expect position-specific and species-specific differences in the intensity of these reactions. Our main objectives are (1) to determine if and to which extent malate and pyruvate influence respiration during light–dark transitions, (2) to identify position- and species-specific differences of both substrates in respiration, (3) and in ^13^C allocation to metabolic fractions. Therefore, we fed different position-specific ^13^C-labeled malate (^13^C-1, ^13^C-4) and pyruvate (^13^C-1, ^13^C-2) via the xylem stream to leaves of two different species, with known high (*Halimium halimifolium*) and low (*Oxalis triangularis*) increases in δ^13^C_LEDR_. We measured the *R*_Label_ during light–dark transitions on-line with high time-resolved isotope laser spectroscopy and determined the ^13^C allocation to leaf metabolic fractions using isotope ratio mass spectrometry.

## Materials and Methods

### Plant Material

We chose species from two functional groups as described in Priault et al. (2009): the evergreen Mediterranean shrub *Halimium halimifolium* L. and the fast-growing herb *Oxalis triangularis* A. St.-Hil. While *H. halimifolium* plants were potted in 5 L pots filled with sand (plant height 40–60 cm), *O. triangularis* plants were potted in 1 L pots with potting soil (plant height 10–15 cm). Both species have a similar LEDR duration, with the main CO_2_-peak declining within approximately 30 min after darkening followed by a slow decrease for another 30 min (Lehmann et al., 2016). All plants were grown under controlled conditions in walk-in climate chambers with a constant temperature of 23°C and relative humidity of about 60% during a 12 h light period (09:00–21:00 h) with a photosynthetic photon flux density (PPFD) of about 770 μmol m^-2^ s^-1^.

### Acquiring of Position-Specific ^13^C-Labeled Malate and Pyruvate

^13^C-1 and ^13^C-4 labeled malate substrates were synthesized with coupled enzymatic reactions modified after Rosenberg and O’Leary (1985): 4.4 mmol NADH disodium salt (Roth, Arlesheim, Switzerland), 2.8 mmol 2-oxoglutarate (Sigma–Aldrich, Buchs, Switzerland), 2.9 mmol ^13^C-1 or ^13^C-4 aspartate (99%, both Sercon, Crewe, UK), and 100 U glutamate-oxaloacetate transaminase (Roche, Rotkreuz, Switzerland) were dissolved in 50 ml of 0.2 M KH_2_PO_4_ (pH 7.5) buffer solution. Subsequently, pH 7.5 was readjusted with 11 ml of 1 M KOH and the reaction solution continuously stirred for 10 min at 25°C. The enzymatic reaction was then started by adding 100 U of a malate dehydrogenase solution (Roche, Rotkreuz, Switzerland). Aliquots of the reaction solution were analyzed at 340 nm with a 96-well microplate reader (ELx800, BioTek, Luzern, Switzerland) to follow the NADH degradation, which ended after 2 h. Thereafter, all enzyme residues of the reaction solution were removed with pre-washed centrifugation filters with a molecular weight cut-off of 5000 da (Vivaspin 15R, 5000 MWCO HY, Sartorius, Göttingen, Germany). Subsequently, aliquots of the reaction solutions were separated by ion exchange chromatography using Dowex material (see below). Malate containing fractions of the reaction solution were eluted from Dowex 1X8 columns with 40 ml 1 M HCl, frozen (-20°C), and lyophilized. Finally, pellets were re-suspended in deionized water and aliquots merged to one labeling solution. Synthesis of ^13^C-1 and ^13^C-4 labeled malate was verified by NMR (Supplementary Figure S1), showing the appropriate position-specific ^13^C-labeling, with only marginal impurities. ^13^C-1 pyruvate (Cambridge Isotope Laboratories, Tewksbury, MA, USA) and ^13^C-2 pyruvate (Sigma-Aldrich, Buchs, Switzerland) were commercially acquired.

### Experimental Setup for Laser-Based On-line Measurements

Leaf ^13^CO_2_ respiration was determined on-line by a cavity ringdown laser spectrometer (CRDS, G2101-I, Picarro, Santa Clara, CA, USA). The CRDS system holds a wavelength monitor, which quantifies the spectral signature of CO_2_ isotopologs with a time-based measurement technique in an optical cavity at 1603 nm. Temperature and pressure within the measurement cavity were controlled at 40°C and 140 Torr, respectively. Data were monitored continuously with a temporal resolution of 0.75 Hz, thus 45 single values were averaged per minute for further analysis.

For all laser-based on-line gas exchange measurements, a transparent 500 ml glass cylinder, with one inlet and one outlet, was used as a plant chamber that enclosed twigs or leaves. Inlets of all plant chambers were flushed continuously with fresh air at a molar mass flow rate of 640 μmol s^-1^. Outlets were connected via Teflon tubing to the CRDS, thus determining ^13^CO_2_ of sample gas (^13^CO_2SG_). For ^13^CO_2_ measurements of reference gas (^13^CO_2RG_), an empty plant chamber of the same size was used. The open bottom side of all plant chambers was sealed with airtight non-fractionating plastic foil (FEP, 4PTFE, Stuhr, Germany). Before and after each ^13^CO_2SG_ measurement of about 40–60 min, ^13^CO_2RG_ was analyzed for about 10–20 min. ^13^CO_2RG_ concentrations (μmol ^13^CO_2_ mol^-1^) were interpolated with a generic regression equation (*y* = *mx* + *b*) for the period of ^13^CO_2SG_ measurements. Switching between ^13^CO_2SG_ and ^13^CO_2RG_ was done manually by re-plugging the CRDS Teflon tubing. ^13^CO_2_ readings were discarded within the first minutes after switching between gases. Compressed air from gas cylinders (4.4 ppm ^13^CO_2_, Riessner-Gase, Lichtenfels, Germany) was analyzed two times per day for about 10–20 min, showing a total variation of *SD* ≤ 0.008 ppm in ^13^CO_2_, thus no significant drift during the experiments occurred.

During the experiments, one twig with leaves of *H. halimifolium* or three leaves of *O. triangularis* still attached to the potted plant were enclosed in the plant chambers. The system was sealed with airtight plastic foil around chamber and stalk of the twigs or leaf petioles. Respiration rates of twigs and leaves were measured in the first 10–20 min to determine the non-labeled leaf ^13^CO_2_ respiration. Subsequently, twigs or leaves within the plant chamber were detached from the plant by cutting and immediately transferred into tap water and cut again under water to prevent air embolism in the xylem. The truncated ends of these twigs and leaves were then directly transferred in reaction vials, which contained the ^13^C-labeling solutions and were placed outside of the plant chamber. The ^13^C-labeling solutions were refilled during the experiments to provide continuously ^13^C-labeled substrates to the plant. Subsequently, ^13^CO_2_ respiration was measured for about 20 min in the light and 20 min in the dark by covering the plant chamber with a light-impermeable cloth. Three to five replicates were measured with each position-specific ^13^C-labeled substrate for each species. Leaf area of all leaves was determined after each experiment.

Tests with dilution series of ^13^C-labeled malate (^13^C-1, ^13^C-4) in *H. halimifolium* and in *O. triangularis* showed that 2 mM malate solutions produced sufficient ^13^CO_2_ emissions and thus this concentration was applied for all further experiments. For ^13^C-labeled pyruvate (^13^C-1, ^13^C-2), 6 mM solutions yielded the best signals. Differences in molar strength by a factor of three for the labeling solutions were accounted for when computing the *R*_Label_ and the Δ_Label_ (see Eqs 1 and 3). All ^13^C-labeled substrates were purely provided without isotopic dilution by unlabeled substrates. No significant changes in total CO_2_ respiration (^12^CO_2_ + ^13^CO_2_) occurred after feeding plants of both species with any position-specific ^13^C-labeled substrate, indicating that the applied concentrations of malate and pyruvate solutions had no significant effect on respiratory CO_2_ emissions. Transpiration rates were monitored by CRDS and checked to be in steady-state during substrate application. Mean transpiration rates during the light in *H. halimifolium* (1.06 ± 0.13 mmol m^-2^ s^-1^) and *O. triangularis* (0.89 ± 0.04 mmol m^-2^ s^-1^) showed no significant differences for all ^13^C-labeled substrates (both species, *p* > 0.05; ANOVA + Tukey-HSD), indicating that absorption rates were similar for all experiments in both species. Air temperature and PPFD within the plant chambers during the measurements was about 29°C and 720 μmol m^-2^ s^-1^, respectively.

Equation 1 was used to calculate *R*_Label_, which was corrected for non-labeled leaf ^13^CO_2_ respiration:

(1) $$R_{\mathrm{Label}}=\left(\frac{f{(}^{13}{\mathrm{CO}}_{2_{\mathrm{SG}}}{-}^{13}{\mathrm{CO}}_{2_{\mathrm{RG}}})}{a}\right)-R_{\mathrm{NL}}$$

where, *f* is the molar mass flow rate (μmol s^-1^), *a* the leaf area (m^2^), ^13^CO_2_SG__ and ^13^CO_2_RG__ the sample and the reference gas (μmol ^13^CO_2_ mol^-1^), and R_NL_ the non-labeled leaf ^13^CO_2_ respiration (μmol ^13^CO_2_ mol^-1^).

Eq. 2 was used to calculate sums of ^13^CO_2_ respiration (∫ _R_) for a distinct period:

(2) $$\int_{R}=\sum_{i=1}^{n}R_{\mathrm{Label}}$$

where *n* is the number of seconds in the light or in the dark.

### Isotopic Analysis of Leaf Metabolic Fractions

Similar to the above-described experiments regarding respiration, an additional experiment was carried out to determine ^13^C allocation of position-specific ^13^C-labeled malate and pyruvate to metabolic fractions. One twig with leaves of *H. halimifolium* or three leaves of *O. triangularis* were placed into reaction vials containing one of the four different position-specific ^13^C-labeled substrates or deionized water to correct for non-labeling conditions. To trace the ^13^C allocation in leaf metabolic fractions during light dark-transitions, leaves of both species were harvested after 20 min in the light, as well as after 40 min (20 min in the light and 20 min in the dark), using additional twigs/leaves of the same plants. Subsequently, leaves from both species were immediately frozen with liquid N_2_, lyophilized, and milled to a fine powder with a ball mill. 100 mg of the plant powder was dissolved in 1.5 ml MCW (methanol, chloroform, water, 12:3:5, v:v:v) and boiled for 30 min at 70°C in a water bath to extract the water soluble fraction, as described in Richter et al. (2009; method I2). Samples were centrifuged for 2 min at 10000 × *g*, and 800 μl of the supernatant were transferred into a new reaction vial. After adding 250 μl chloroform, samples were shaken intensively and centrifuged for 2 min at 10000 × *g*. For the isotopic analysis of lipids, aliquots of the lower chloroform phase were carefully taken to avoid contamination with the upper phase and transferred into tin capsules. In a next step, 1.2 ml of the upper phase was transferred into a new reaction vial, mixed roughly with 500 μl chloroform, and centrifuged 2 min at 10000 × *g*. Finally, 1 ml of the upper phase, which contained the total hydrophilic fraction of the extract, was transferred into a new reaction vial and stored at -20°C.

The hydrophilic fraction was further separated to sugar, amino acid, and organic acid fractions with ion-exchange chromatography using Dowex material (100–200 mesh, Sigma–Aldrich, Buchs, Switzerland; see Richter et al., 2009). In brief, different Dowex materials were conditioned with 1M HCL (Dowex 50WX8) or with 1M sodium formate (Dowex 1X8) and transferred to columns, which were placed above each other on a rack. Columns were extensively prewashed with deionized water to fully remove potential carbon residues. Subsequently, the hydrophilic fraction was added and the neutral, sugar containing fraction was eluted with 35 ml deionized water, while the flow-through was collected in 50 ml falcon tubes. The amino acid fraction was absorbed by Dowex 50WX8 and eluted with 30 ml 3M NH_3_, while the organic acid fraction was absorbed by Dowex 1X8 and eluted with 35 ml 1M HCL. Eluates of all three fractions were frozen at -20°C, lyophilized, and the pellets re-suspended in deionized water. Subsequently, aliquots of the fractions were transferred into tin capsules. All capsules (including lipids) were oven-dried at 60°C and δ^13^C values analyzed with an EA-IRMS (Thermo Fisher Scientific, Bremen, Germany). Measurements and referencing for the IRMS were done after Werner et al. (1999) and Werner and Brand (2001). The IRMS long-term precision of a quality control standard (-43.11‰, Caffeine, Fluka, Buchs, Switzerland) was ≤0.15‰ (*SD*).

Equation 3 was used to calculate the Δ_Label_ in leaf metabolic fractions, which was corrected for δ^13^C values under non-labeling conditions:

(3) $$\Delta_{\mathrm{Label}}=\;\delta^{13}C_{L}-\;\delta^{13}C_{\mathrm{NL}}$$

where δ^13^C_L_ are the δ^13^C values in leaf metabolic fractions (lipids, sugars, amino and organic acids) of plants fed with position-specific ^13^C-labeled substrates, and δ^13^C_NL_ is the mean δ^13^C value of the corresponding leaf metabolic fraction under non-labeling conditions.

### Statistics

General linear models were performed to test for position-specific, species-specific, and temporal differences in ∫ _R_ and Δ_Label_ of different metabolic fractions. One-way analysis of variance (ANOVA) and Tukey-HSD *post hoc* tests were used to show differences in Δ_Label_ of different metabolic fractions for each ^13^C-labeled substrate. Unless otherwise specified, means and standard errors are given. All statistical analyses were performed in R (version 3.1.3; R Core Team, 2015).

## Results

### Leaf ^13^CO_2_ Respiration of Different Position-Specific ^13^C-Labeled Substrates and Species

To identify the respiratory substrates and enzymatic reactions, determining the species-specific differences in δ^13^C_LEDR_ at natural isotope abundances in *H. halimifolium* (high increases in δ^13^C_LEDR_) and in *O. triangularis* (low increases in δ^13^C_LEDR_), we fed those plants with different ^13^C-labeled malate and pyruvate substrates, expecting position- and species-specific differences in ^13^CO_2_ respiration during light–dark transitions.

**FIGURE 1:**
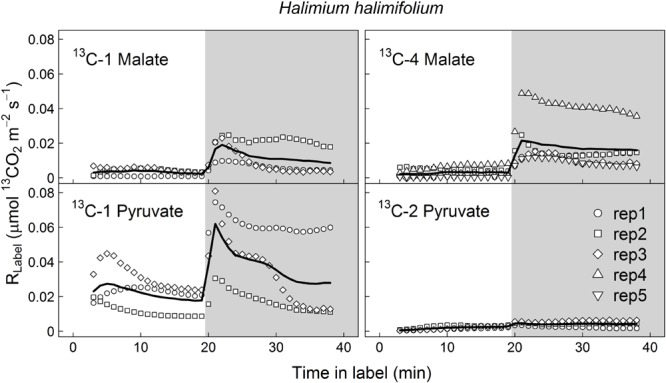
**Label-derived leaf ^13^CO_2_ respiration (*R*_Label_, μmol ^13^CO_2_ m^-2^ s^-1^) during light–dark transitions in *Halimium halimifolium*.** Twigs with leaves were fed with position-specific ^13^C-labeled malate (^13^C-1, ^13^C-4) or pyruvate (^13^C-1, ^13^C-2) for about 40 min (20 min in the light and 20 min in the dark). Gray areas denote dark periods. Black bold lines denote mean values, while symbols indicate single replicates (*n* = 3–5 individuals).

**FIGURE 2:**
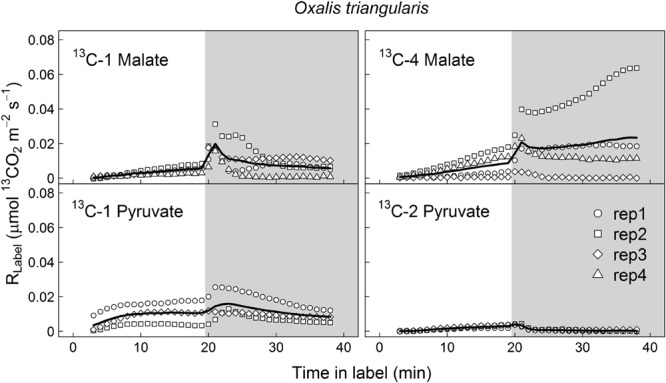
**Label-derived leaf ^13^CO_2_ respiration (*R*_Label_, μmol ^13^CO_2_ m^-2^ s^-1^) during light–dark transitions in *Oxalis triangularis*.** Leaves were fed with position-specific ^13^C-labeled malate (^13^C-1, ^13^C-4) or pyruvate (^13^C-1, ^13^C-2) for about 40 min (20 min in the light and 20 min in the dark). Gray areas denote dark periods. Black bold lines denote mean values, while symbols indicate single replicates (*n* = 3–4 individuals).

^13^CO_2_ respiration rates (*R*_Label_) from ^13^C-1 and ^13^C-4 malate were low in the light, but steeply increased shortly after darkening during LEDR, with a peak of about 0.02 μmol ^13^CO_2_ m^-2^ s^-1^ (**Figures 1** and **2**). Clear light–dark differences for sums of the ^13^CO_2_ respiration rates (∫ *_R_*) reflect the observed *R*_Label_ patterns (**Tables 1** and **2**). Furthermore, ∫ *_R_* of malate substrates showed no significant position-specific and species-specific difference, showing that ^13^CO_2_ respiration from ^13^C-1 malate did not differ significantly compared to ^13^CO_2_ respiration from ^13^C-4 malate in both species during light–dark transitions (**Tables 1** and **2**).

**Table 1:** Sums of ^13^CO_2_ respiration (∫ *_R_*) derived from different ^13^C-labeled substrates.

Species	Label	∫ *_R_*_-light_	∫ *_R_*_-dark_
*Halimium halimifolium*	^13^C-1 Malate	0.06 ± 0.02	0.22 ± 0.08
	^13^C-4 Malate	0.05 ± 0.02	0.33 ± 0.11
	^13^C-1 Pyruvate	0.37 ± 0.10	0.71 ± 0.24
	^13^C-2 Pyruvate	0.03 ± 0.01	0.08 ± 0.02
*Oxalis triangularis*	^13^C-1 Malate	0.05 ± 0.01	0.17 ± 0.05
	^13^C-4 Malate	0.08 ± 0.03	0.38 ± 0.19
	^13^C-1 Pyruvate	0.16 ± 0.06	0.23 ± 0.07
	^13^C-2 Pyruvate	0.03 ± 0.00	0.02 ± 0.00

*∫ _*R*_ values from different ^13^C-labeled malate and pyruvate substrates were calculated for light (∫ *_R-light_*, μmol ^13^CO_2_ m^-2^ s^-1^) and dark periods (∫ _*R-dark*_, μmol ^13^CO_2_ m^-2^ s^-1^) in *Halimium halimifolium* and *Oxalis triangularis* plants. Means and SE are given (*n* = 3–5 individuals). Please refer to **Table 2** for statistical analyses.*

**Table 2:** Statistical analyses of ∫ *_R_* and Δ_Label_ values from different metabolic fractions.

Parameter	∫ *_R_*	Lipids	Sugars	Organic Acids	Amino Acids
Malate	0.191	0.163	0.764	0.860	0.094
Species	0.960	**0.001**	NA	**0.007**	**0.032**
Malate^∗^Species	0.614	0.117	NA	0.666	0.240
Time	**0.001**	0.138	0.902	0.142	0.537

Pyruvate	**0.001**	**0.001**	0.065	**0.004**	0.273
Species	**0.015**	**0.001**	NA	**0.001**	0.782
Pyruvate^∗^Species	**0.039**	0.544	NA	0.068	0.183
Time	0.129	**0.028**	0.925	0.101	0.494

*Results of general linear models show light–dark (Time), position-specific (Malate, Pyruvate), and species-specific (Species) differences and their interactions (e.g., Malate^∗^Species). Please note that we separately performed statistical tests on malate and pyruvate, since a comparison of the absolute ∫ _*R*_ and Δ_*Label*_ values from both substrates is potentially biased by differences in substrate transport from the assimilation site to the site of reaction, mixing with leaf internal substrate pools, and substrate compartmentalization. *P*-values are indicated. Significant values (*P* ≤ 0.05) are marked in bold.*

In contrast to both ^13^C-labeled malate substrates, which can be released by different reactions (NAD-ME, TCA cycle), ^13^CO_2_ respiration rates from ^13^C-1 pyruvate reflect the direct CO_2_ release by a pyruvate dehydrogenase reaction (cpPDH or mtPDH). Highest *R*_Label_ values from ^13^C-1 pyruvate were observed in *H. halimifolium*, with about 0.025 μmol ^13^CO_2_ m^-2^ s^-1^ in the light, and with a pronounced peak of 0.06 μmol ^13^CO_2_ m^-2^ s^-1^ within the first minutes in the dark during LEDR, which was followed by a clear continuous decrease (**Figure 1**). A very different ^13^CO_2_ respiration pattern from ^13^C-1 pyruvate was found in *O. triangularis*, with no clear peak during LEDR (**Figure 2**). *R*_Label_ from ^13^C-2 pyruvate, reflecting the activity of CO_2_ releasing reactions within the TCA cycle, showed hardly any increase in both species (**Figures 1** and **2**). Statistical analyses on ∫ *_R_* values are in agreement with the observed ^13^CO_2_ respiration patterns from pyruvate substrates, showing a species-dependency for changes caused by the ^13^C-labeled substrates and no clear light–dark differences (**Tables 1** and **2**). A Tukey HSD *post hoc* test revealed significant position-specific differences for ∫ *_R_* from pyruvate substrates during light–dark transitions in *H. halimifolium* (*P* < 0.001), but not in *O. triangularis* (*P* = 0.354). It also shows species-specific differences for ∫ *_R_* from ^13^C-1 pyruvate (*P* ≤ 0.012), but not for ∫ *_R_* from ^13^C-2 pyruvate (*P* = 0.987). In summary, clear position- and species-specific differences during LEDR were only observed in the ^13^CO_2_ respiration pattern obtained from pyruvate.

### ^13^C Allocation to Leaf Metabolic Fractions

Carbon isotope ratios in leaf metabolic fractions (lipids, sugars, amino and organic acids) of *H. halimifolium* and *O. triangularis* plants did not show significant light–dark differences between the two sampling time points, except for lipids from plants fed with ^13^C-2 pyruvate (**Table 2**). Thus, mean values of both time points were used for further analysis, i.e., to determine ^13^C allocation of position-specific ^13^C-labeled substrates to metabolic fractions.

**FIGURE 3:**
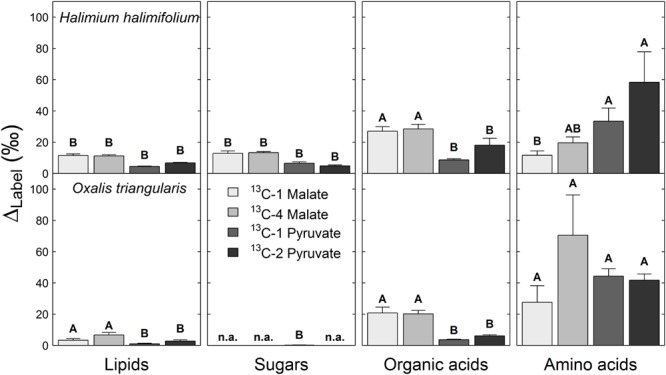
**^13^C allocation of position-specific^13^C-labeled malate (^13^C-1, ^13^C-4) and pyruvate (^13^C-1, ^13^C-2) to metabolic fractions in *Halimium halimifolium* and *Oxalis triangularis*.** The label-induced ^13^C increase corrected for non-labeling conditions (Δ_Label_, ‰) indicates the amount of total ^13^C allocated to leaf metabolic fractions. Mean Δ_Label_ values for the two harvests (20 min light or 20 min light and 20 min dark) were calculated, since no significant light–dark differences were observed for Δ_Label_, except for lipids in plants fed with ^13^C-2 pyruvate, which showed higher δ^13^C values in the dark than in the light **(**Table 2**)**. Please refer to **Table 2** for the statistical analyses. n.a. indicates no observable Δ_Label_. Capital letters denote significant differences among metabolic fractions of plants fed with the same ^13^C-labeled substrate (ANOVA and Tukey-HSD). Means and SE are given (*n* = 3 individuals).

The Δ_Label_ derived from both ^13^C-labeled pyruvate substrates was highest in the amino acid fraction of both species, while Δ_Label_ derived from ^13^C-labeled malate substrates was found to be highest in both, the amino acid and the organic acid fractions (**Figure 3**). In contrast, Δ_Label_ derived from any ^13^C-labeled substrate was lowest in lipid and sugar fractions of in both species, or showed no distinct ^13^C allocation. Significant position-specific differences were only found in lipid and organic acid fractions derived from ^13^C-labeled pyruvate substrates, with higher Δ_Label_ values from ^13^C-2 pyruvate compared to those from ^13^C-1 pyruvate (**Table 2**). Significant species-specific differences were observed across different ^13^C-labeled substrates and fractions. In summary, ^13^C from malate and pyruvate was species-specifically allocated to leaf metabolic fractions, mainly to amino and organic acids under light conditions, while position-specific differences in ^13^C allocation were only observed for pyruvate.

## Discussion

### Carboxyl Groups of Malate Similarly Fuel Respiration during Light–Dark Transitions

**FIGURE 4:**
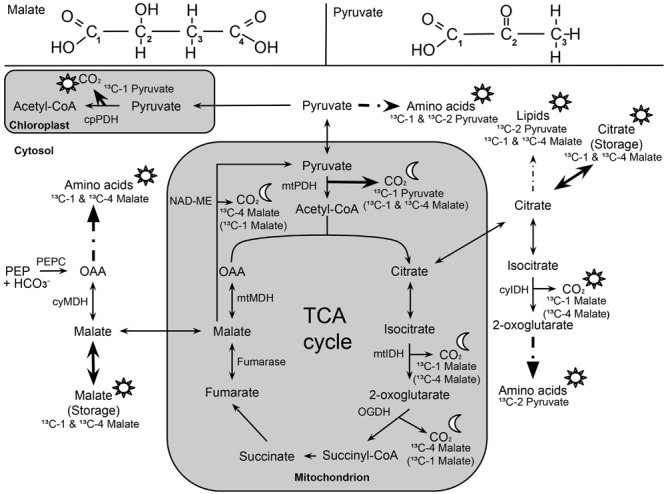
**Overview of ^13^C allocation from position-specific ^13^C-labeled malate (^13^C-1, ^13^C-4) and pyruvate (^13^C-1; ^13^C-2) during light–dark transitions.** Sun (☼) and half-moon (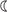) symbols denote temporary ^13^C allocation during light or dark periods (LEDR), respectively. Solid arrows indicate ^13^C allocation of highest importance, while dashed arrows indicate reactions with intermediate steps. ^13^C-labeled malate substrates in brackets denote alternative respiratory pathways due to the influence of NAD-ME (decarboxylation of C-4 position of malate) and fumarase (randomization of C-1 and C-4 position of malate). The following abbreviations are used: cyIDH and mtIDH, NADP-cytosolic and NAD-mitochondrial isocitrate dehydrogenase; cyMDH and mtMDH, cytosolic and mitochondrial malate dehydrogenase; NAD-ME, NAD-malic enzyme; OAA, oxaloacetate; OGDH, 2-oxoglutarate dehydrogenase; cpPDH and mtPDH, chloroplast and mitochondrial pyruvate dehydrogenase; PEPC, phosphoenolpyruvate carboxylase; TCA cycle, tricarboxylic acid cycle.

Here, we investigated the ^13^CO_2_ respiration pattern from different position-specific ^13^C-labeled malate and pyruvate in species with marked differences in δ^13^C_LEDR_ to identify potential respiratory substrates and enzymatic reactions. Both, ^13^C-1 and ^13^C-4 malate were clearly respired during the light–dark transitions in *H. halimifolium* and *O. triangularis* (**Figures 1** and **2**; **Table 1**), demonstrating that both carboxyl groups of malate contribute to respiration, especially during LEDR. Interestingly, the ^13^CO_2_ respiration rates from ^13^C-1 and ^13^C-4 malate were similar in both species, which is surprising since only the C-4 position of malate have been suggested to be ^13^C enriched compared to other molecule positions (Melzer and O’Leary, 1987; Savidge and Blair, 2004) and thus expected to be of high importance for the ^13^C enrichment in respired CO_2_ during LEDR at natural isotope abundances (Barbour et al., 2007; Gessler et al., 2009; Werner et al., 2011; Tcherkez et al., 2012). The equal contribution of both carboxyl groups of malate to respiration in both species is most likely provoked by the fumarase activity, causing a partial isotope randomization of the carboxyl groups of malate (Bradbeer et al., 1958; Gout et al., 1993). For instance, ^13^C-label at the C-4 position of malate can be transferred to the C-1 position of malate, which can be decarboxylated by NAD-ME, producing ^13^C-1 labeled pyruvate that fuels mtPDH (**Figure 4**). This respiratory flux is in line with high NAD-ME activity during LEDR (Igamberdiev et al., 2001), which may be stimulated by fumarate in support of oxidation of photosynthetic substrates (Tronconi et al., 2015). Moreover, if randomization of malate by the fumarase reaction is assumed, the ^13^C-label from both carboxyl groups of malate could be released by the action of the NAD-dependent mtIDH and the OGDH in the TCA cycle. Otherwise, these reactions would only release one carboxyl group of malate; C-1 by mtIDH and C-4 by OGDH (**Figure 4**).

Decarboxylation of ^13^C-labeled malate in the light was generally not very strong, but also visible in all species (**Figures 1** and **2**; **Table 1**). The theoretical background for malate respiration in light was previously described by Werner et al. (2011). At least for ^13^C-1 malate, this might be explained by conversion of malate via the mtMDH reaction to oxaloacetate, which is used together with glycolytic acetyl-CoA by the citrate synthase reaction to build up citrate. This citrate can then be transferred to the cytosol via the citrate shuttle, where it is converted to isocitrate and subsequently decarboxylated to α-ketoglutarate and CO_2_ by the NADP-dependent cytosolic isocitrate dehydrogenase reaction (cyIDH, **Figure 4**) (Werner et al., 2011). On the other hand, less is known about the biochemical pathways for direct respiration of ^13^C-4 malate in the light, since NAD-ME (Hill and Bryce, 1992; Igamberdiev et al., 2001) and TCA cycle reactions (Hanning and Heldt, 1993; Tcherkez et al., 2005; Araújo et al., 2012) are known to be partially down-regulated. Cytosolic fumarase might be involved, which was found to be expressed in some species (Pracharoenwattana et al., 2010; Eprintsev et al., 2014). However, the role of the enzyme in malate randomization in the light and during LEDR has not been elucidated yet.

### Position- and Species-Specific Differences in Pyruvate Respiration Determine Natural δ^13^C_LEDR_ Patterns

In contrast to ^13^C-labeled malate substrates, position-specific differences in *R*_Label_ were induced by the ^13^C-labeled pyruvate substrates (**Figures 1** and **2**; **Table 1**), which were, however, species-dependent (**Table 2**). Differences in ^13^CO_2_ respiration from ^13^C-1 pyruvate and ^13^C-2 pyruvate were significant in *H. halimifolium*, the species with high natural increases in δ^13^C_LEDR_ (up to 14.8‰; Wegener et al., 2010), but not in *O. triangularis*, the species which exhibits only low natural increases in δ^13^C_LEDR_ (up to 3.4 ‰; Wegener et al., 2010). This demonstrates a biochemical link between respiration of the C-1 position of pyruvate and the known increases in δ^13^C_LEDR_ and suggests a higher mtPDH activity during LEDR in *H. halimifolium* compared to *O. triangularis* (Priault et al., 2009; Wegener et al., 2010). Pyruvate for the mtPDH reaction may be provided by different fluxes, e.g., by glycolysis, by anaplerotic PEPC fluxes (via PEPC, MDH, NAD-ME), and by fluxes from carbon storage pools such as citrate and malate (via decarboxylation reactions from TCA cycle and NAD-ME; **Figure 4**).

We also observed an increase in ^13^CO_2_ respiration rates from ^13^C-1 pyruvate in the light, especially in *H. halimifolium* (**Figures 1** and **2**; **Table 1**). This was most likely mainly caused by the chloroplast PDH reaction (cpPDH, **Figure 4**; Tcherkez et al., 2005, 2012), since the mitochondrial PDH reaction is known to be strongly down regulated under illumination (mtPDH, **Figure 4**), (Budde and Randall, 1990). In contrast, ^13^C-2 pyruvate was only weakly respired during the light–dark transitions in both species (**Figures 1** and **2**; **Table 1**). This suggests that the C-2 position of pyruvate (reflecting the acetyl-CoA residue) is predominantly used for biosynthesis of compounds during light–dark transitions. Indeed, this was further supported by position-specific ^13^C allocation differences in leaf metabolic fractions originated from ^13^C-labeled pyruvate substrates as described in the next paragraph.

### ^13^C-Label from Malate and Pyruvate Is Mainly Allocated to Amino and Organic Acids

Our final objective was to determine the ^13^C allocation from position-specific ^13^C-labeled substrates to metabolic fractions. We typically observed that ^13^C allocation to metabolic fractions (lipids, sugars, amino and organic acids) was high under light conditions, but immediately decreased when plants were transferred into darkness. This observation can be explained by well-known changes in transpiration and respiration rates in the dark. On the one hand, darkening causes a decrease of transpiration rates (due to decrease in vapor pressure deficit) and thus lower substrate uptake and less ^13^C allocation to metabolic fractions. On the other hand, most of the ^13^C-labeled substrates are used for respiration rather than for biosynthesis in the dark, causing an increase in ^13^CO_2_ respiration rates during LEDR in both species (**Figures 1** and **2**). With one exception, lipids in plants fed with ^13^C-2 pyruvate were significantly more enriched in ^13^C after darkening (**Figure 3**; **Table 2**), suggesting that the acetyl-CoA produced from pyruvate is especially used for lipogenesis during light–dark transitions. This also partially explains the low ^13^CO_2_ respiration rates from ^13^C-2 pyruvate compared to those from ^13^C-1 pyruvate in both species (**Figures 1** and **2**).

Furthermore, our results show that the ^13^C-label from both malate and pyruvate were generally allocated to the amino and organic acid fractions in both species. As summarized in **Figure 4**, the substrates were mostly used as precursors for the synthesis of amino acids derived from pyruvate, oxaloacetate, 2-oxoglutarate, as well as for the synthesis of organic acids such as citrate and malate, which are TCA cycle intermediates (Werner et al., 2011) or used as carbon storage molecules (Zell et al., 2010). However, with respect to the ^13^C-labeled substrates, we cannot distinguish if the organic acids taken up via the xylem stream were fully used for biosynthesis or if they partly accumulated in the leaves. Δ_Label_ values in lipids for both species suggested that only small amounts of the position-specific ^13^C-labeled malate and pyruvate were used for lipogenesis (**Figures 3** and **4**); similar results was found in a recent ^14^C labeling study (Grimberg, 2014). Low Δ_Label_ values in sugars in *H. halimifolium* might be explained by photosynthetic fixation of respired CO_2_ derived from the ^13^C-labeled substrates, as previously observed for pyruvate (Tcherkez et al., 2005) or malate substrates (Hibberd and Quick, 2002).

## Conclusion

Based on our experiments, we provide first direct experimental evidence that both carboxyl groups of malate are used as respiratory substrates during LEDR in different species. Surprisingly, plants fed with ^13^C-1 or ^13^C-4 malate showed no position- and species-specific differences in respiration pattern and metabolic fractions, strongly indicating that the carboxyl groups of malate undergo leaf internal isotope randomization by fumarase. Thus, our findings introduce a new level of complexity to the interpretation of respiratory substrates and enzymatic reactions influencing δ^13^C of leaf-respired CO_2_ at natural isotope abundances. We conclude that malate respiration via different enzymatic reactions and metabolic pathways (e.g., fumarase, NAD-ME, and TCA cycle) supports and supplies pyruvate respiration during LEDR, which determines the species-specific differences of natural δ^13^C_LEDR_ patterns via decarboxylation of the C-1 position of pyruvate by PDH reactions, as shown within this study.

## Author Contributions

ML and FW equally contributed to the manuscript. ML, FW, CW, and RW designed the experiments. ML synthesized the position-specific ^13^C-labeled malate. FW assembled the experimental setup. ML and FW performed the experiments and acquired the raw data. ML, FW, and MB processed the data. RW, CW, NB, RS, and VM supervised data analyses and interpretation. All authors helped drafting the manuscript and gave essential input to the work.

## Conflict of Interest Statement

The authors declare that the research was conducted in the absence of any commercial or financial relationships that could be construed as a potential conflict of interest.

## Abbreviations

δ^13^C_LEDR_: carbon isotopic composition of leaf-respired CO_2_ during LEDR
Δ_Label_: label-induced ^13^C increase
cyIDH and mtIDH: cytosolic and mitochondrial isocitrate dehydrogenase
LEDR: light enhanced dark respiration
cyMDH and mtMDH: cytosolic and mitochondrial malate dehydrogenase
NAD-ME: NAD-malic enzyme
OGDH: 2-oxoglutarate dehydrogenase
cpPDH and mtPDH: chloroplast and mitochondrial pyruvate dehydrogenase
PEPC: phosphoenolpyruvate carboxylase
R_Label_: label-derived leaf ^13^CO_2_ respiration
TCA cycle: tricarboxylic acid cycle
