# Supplementary material for: Metabolic Fate of the Carboxyl Groups of Malate and Pyruvate and their Influence on δ13C of Leaf-Respired CO2 during Light Enhanced Dark Respiration
Source: Front Plant Sci. 2016 Jun 3;7:739. doi: 10.3389/fpls.2016.00739 (PMC4891945; doi:10.3389/fpls.2016.00739)
Supplement: Supplementary file 1 [file Image_1.PDF]

Supplementary Material

Metabolic Fate of the Carboxyl Groups of Malate and Pyruvate and Their Influence on $\delta^{13}C$ of Leaf Respired CO_2 during Light Enhanced Dark Respiration

Marco M. Lehmann[*,†], Frederik Wegener[†], Matti Barthel, Veronica G. Maurino, Rolf T.W. Siegwolf, Nina Buchmann, Christiane Werner, Roland A. Werner

[†] **Authors equally contributed to the manuscript**

*** Corresponding Author:** marco.lehmann@alumni.ethz.ch

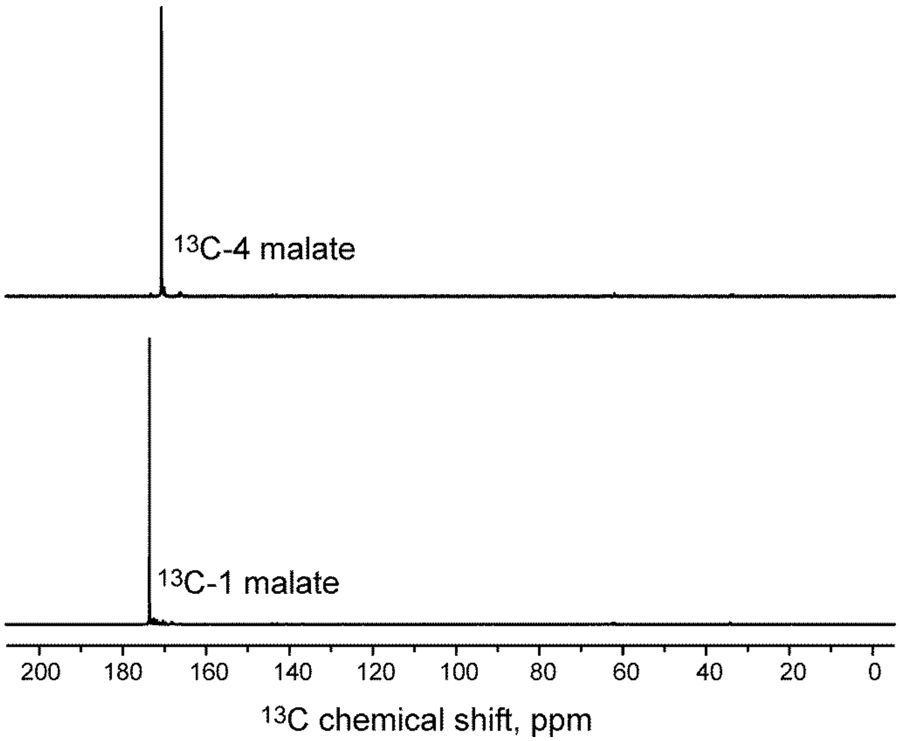

Supplementary Figure 1: NMR analysis of ^{13}C-1 and ^{13}C-4 labelled malate.
